# Supplementary material for: Effect of Serum Lipid Profile on the Risk of Breast Cancer: Systematic Review and Meta-Analysis of 1,628,871 Women
Source: J Clin Med. 2022 Aug 2;11(15):4503. doi: 10.3390/jcm11154503 (PMC9369572; doi:10.3390/jcm11154503)
Supplement: Supplementary file 1 [file jcm-11-04503-s001.zip › jcm-1729587-supplementary.pdf]

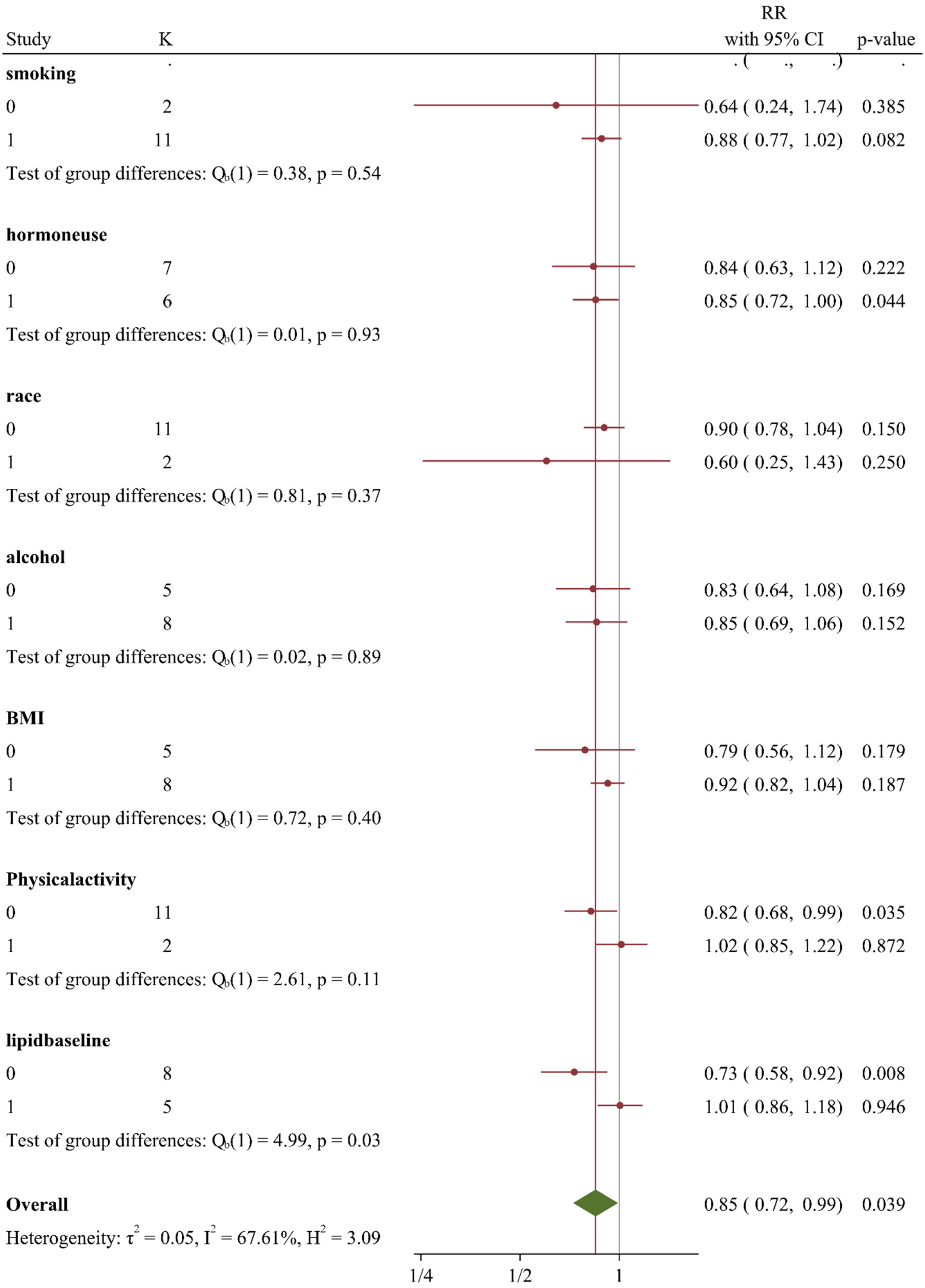

Figure S1. Subgroup analysis for HDL-C.

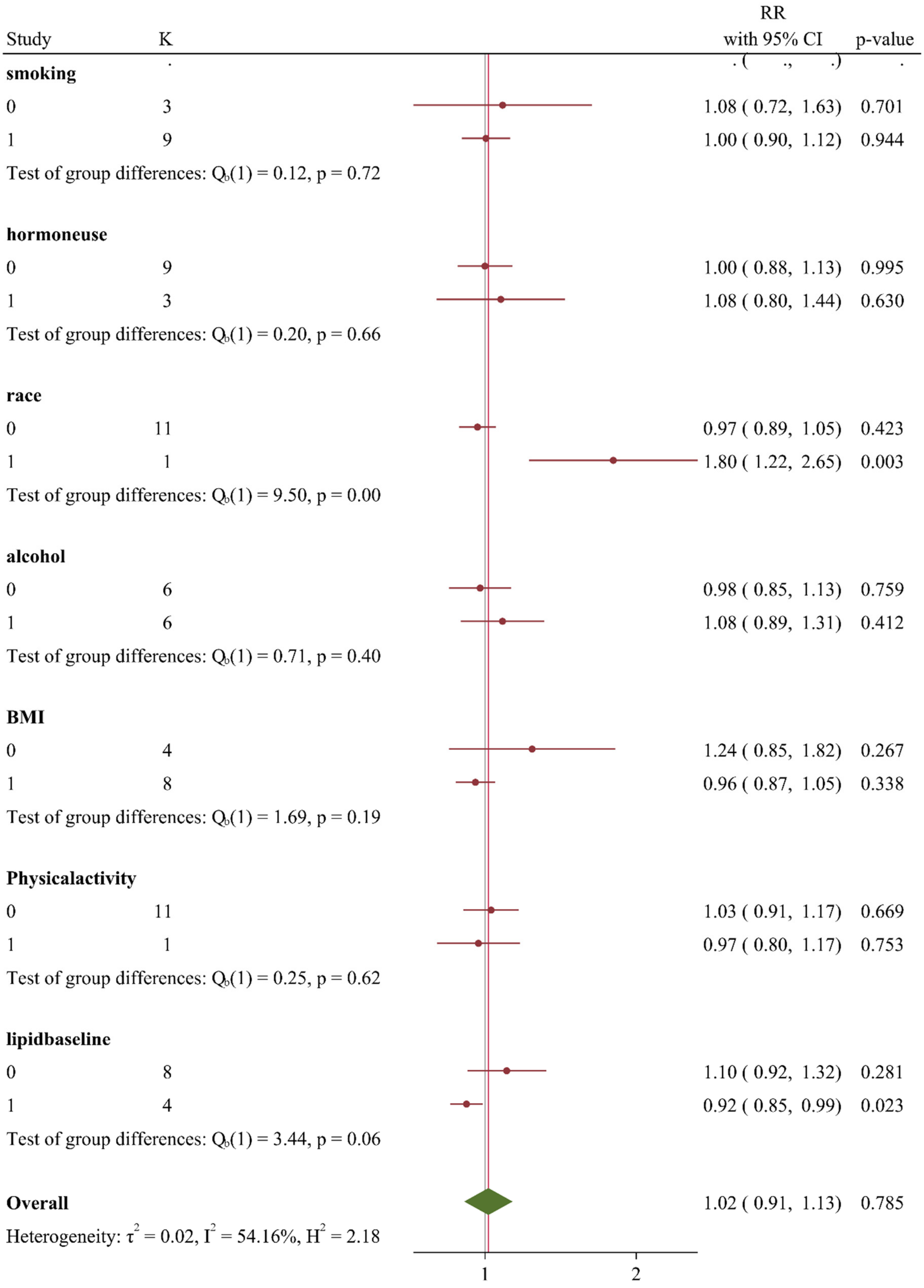

Figure S2. Subgroup analysis for triglyceride.

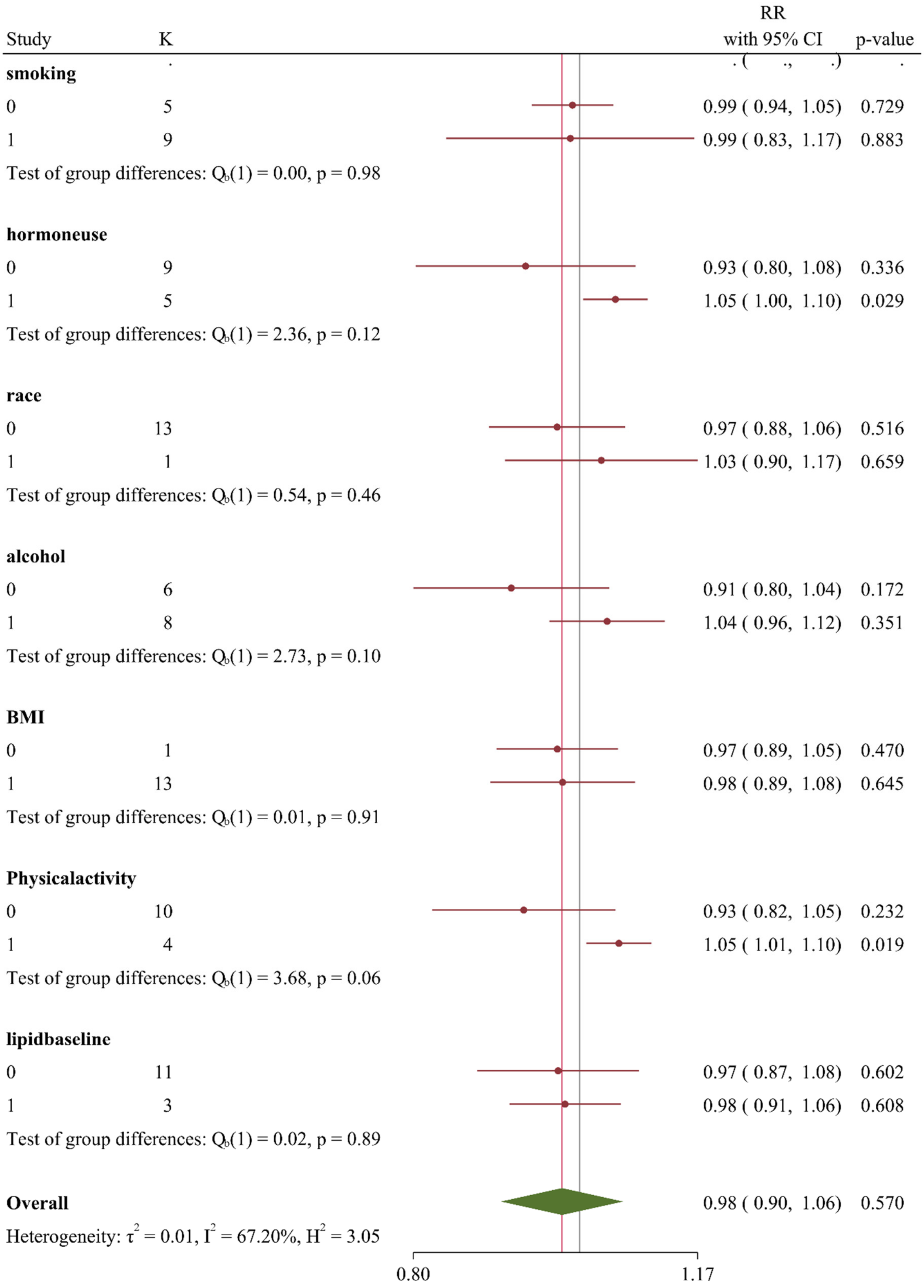

Figure S3. Subgroup analysis for total cholesterol.
